# Hypertension and Genetic Variation in Endothelial-Specific Genes

**DOI:** 10.1371/journal.pone.0062035

**Published:** 2013-04-29

**Authors:** Erik Larsson, Björn Wahlstrand, Bo Hedblad, Thomas Hedner, Sverre E. Kjeldsen, Olle Melander, Per Lindahl

**Affiliations:** 1 Department of Medical Biochemistry and Cell Biology, Institute of Biomedicine, The Sahlgrenska Academy at University of Gothenburg, Gothenburg, Sweden; 2 Institute of Medicine, The Sahlgrenska Academy at University of Gothenburg, Gothenburg, Sweden; 3 Clinical research Center (CRC), Malmö University Hospital, Malmö, Sweden; 4 Department of Cardiology/Cardiovascular and Renal Research Center, Ullevaal University Hospital, Oslo, Norway; 5 Wallenberg Laboratory, Institute of Medicine, The Sahlgrenska Academy at University of Gothenburg, Gothenburg, Sweden; Medical University Hamburg, University Heart Center, Germany

## Abstract

Genome-wide association (GWA) studies usually detect common genetic variants with low-to-medium effect sizes. Many contributing variants are not revealed, since they fail to reach significance after strong correction for multiple comparisons. The WTCCC study for hypertension, for example, failed to identify genome-wide significant associations. We hypothesized that genetic variation in genes expressed specifically in the endothelium may be important for hypertension development. Results from the WTCCC study were combined with previously published gene expression data from mice to specifically investigate SNPs located within endothelial-specific genes, bypassing the requirement for genome-wide significance. Six SNPs from the WTCCC study were selected for independent replication in 5205 hypertensive patients and 5320 population-based controls, and successively in a cohort of 16537 individuals. A common variant (rs10860812) in the DRAM (damage-regulated autophagy modulator) locus showed association with hypertension (*P* = 0.008) in the replication study. The minor allele (A) had a protective effect (OR = 0.93; 95% CI 0.88–0.98 per A-allele), which replicates the association in the WTCCC GWA study. However, a second follow-up, in the larger cohort, failed to reveal an association with blood pressure. We further tested the endothelial-specific genes for co-localization with a panel of newly discovered SNPs from large meta-GWAS on hypertension or blood pressure. There was no significant overlap between those genes and hypertension or blood pressure loci. The result does not support the hypothesis that genetic variation in genes expressed in endothelium plays an important role for hypertension development. Moreover, the discordant association of rs10860812 with blood pressure in the case control study *versus* the larger Malmö Preventive Project–study highlights the importance of rigorous replication in multiple large independent studies.

## Introduction

Hypertension is the major global risk factor for coronary heart disease and stroke. The pathogenesis is poorly understood and the primary cause is unknown in 90–95% of cases. Heritability has been estimated to between 30 and 50% and ambitious efforts have been made to elucidate the genetic basis. While genome-wide association (GWA) studies have been effective at identifying novel genetic risk loci for a wide range of diseases, hypertension has proved more challenging. The Welcome Trust Case Control Consortium (WTCCC) study [1], encompassing 2000 subjects for each of 7 diseases and 3000 common controls, was successful overall but failed in the case of hypertension. Robust associations of genetic variants with hypertension were eventually identified based on large meta-analyses and multi-center studies of 30,000–200,000 individuals [2]–[6].

One possible reason for this is that hypertension may have relatively few common risk alleles of large effect sizes [7]. Thus, discovery of novel variants for hypertension on the whole genome scale is challenging, and many true variants are likely fail to reach significance after correction for multiple testing. Although no single nucleotide polymorphisms (SNPs) reached the P<5×10^−7^ threshold required for significance in the WTCCC hypertension study, the number and distribution of signals in the 10^−7^ to 10^−4^ range was similar to that observed for other diseases [1]. It is therefore possible that several truly hypertension-associated variations are hidden among the long list of moderately significant SNPs.

**Figure 1:**
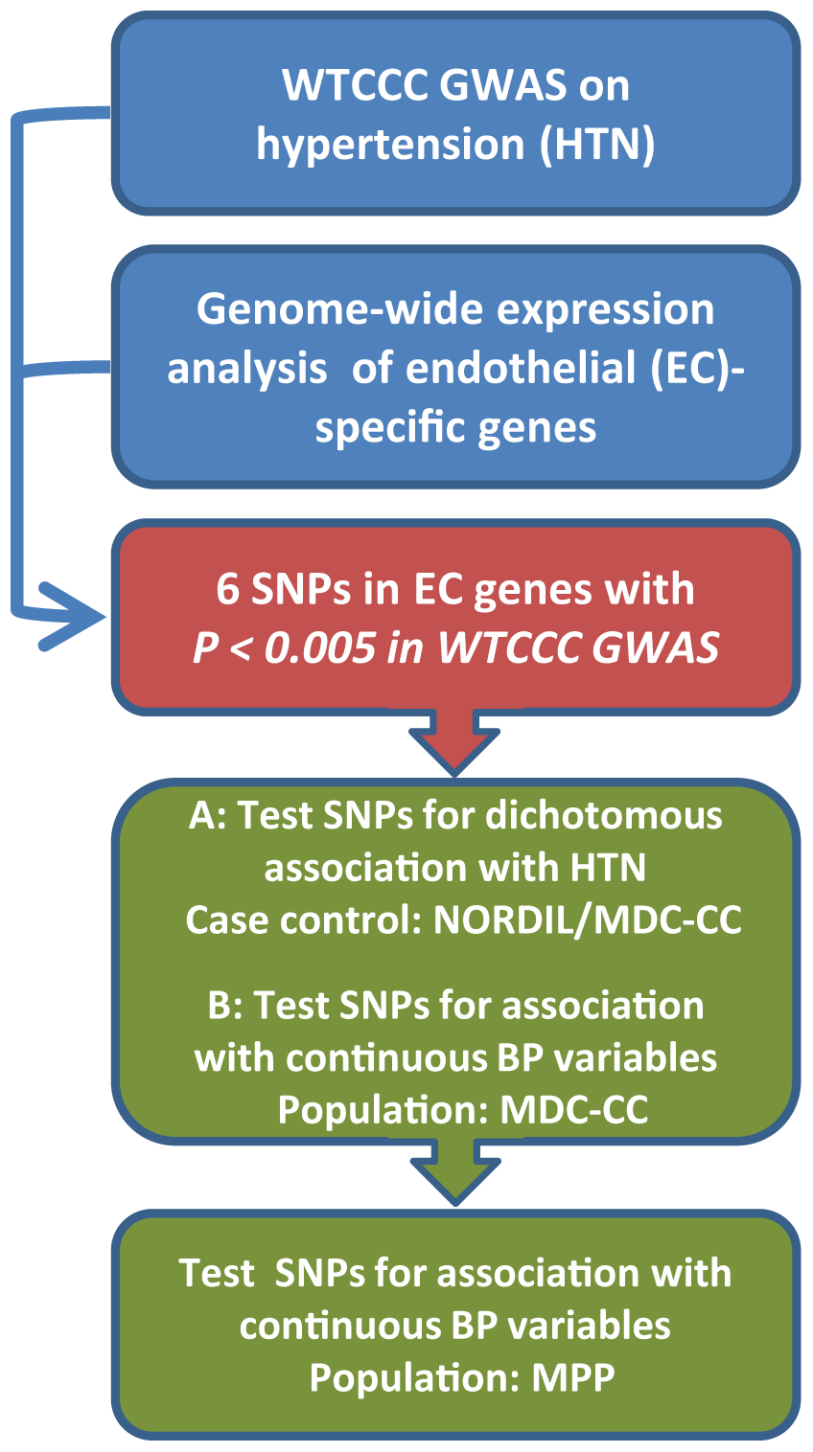
Study design and workflow.

Although results have been contradictive, previous association studies indicate that genetic variation in endothelial genes such as endothelin-1 and endothelial nitric oxide synthase, both related to blood pressure (BP) regulation, may contribute to essential hypertension [8]–[11]. Based on the hypothesis that genetic variation in endothelium-specific genes may influence BP, we combined data from the WTCCC GWA study with previously published microarray gene expression data from mice [12], to select a subset of six SNPs, all located within endothelial marker genes, for independent replication in a case control study comprising more than 10,000 individuals. One SNP was further evaluated in a population-based study with 16537 participants (Figure 1).

## Methods

### Ethics Statement

The Nordic Diltiazem study (NORDIL) and the Malmö Diet and Cancer Cardiovascular Cohort study (MDC-CC) were approved by the human ethics committees at the University of Gothenburg and Lund University, respectively. The Malmö Preventive Project (MPP) study was approved by the local ethics committee of Southern Sweden. All study participants had given written consent.

### The Nordic Diltiazem Study

The Nordic Diltiazem study (NORDIL) is an intervention trial that between 1992 and 1999 included 10881 Swedish and Norwegian patients diagnosed with severe (grade 2) hypertension based on repeated diastolic BP≥100 mmHg on different occasions, and prospectively compared cardiovascular outcome in patients randomized to diltiazem-based antihypertensive treatment as compared to patients randomized to diuretic and/or β-blocker based antihypertensive treatment. The design and main results have previously been described in detail [13]. The primary endpoint was fatal and non-fatal stroke, fatal and non-fatal myocardial infarction and other cardiovascular deaths, and there was no significant difference in cardiovascular outcome between the two treatment regimens. The Swedish subcohort of NORDIL participated in a genetic study and we obtained whole blood samples for DNA isolation from 5262 patients, of whom 5205 were successfully genotyped for the main SNP rs10860812 for the present investigation. BP was measured in the supine position after 10 minutes rest. These patients with diagnosed grade 2 hypertension formed our case group.

### The Malmö Diet and Cancer Cardiovascular Cohort study

As a population control we used a Swedish cohort study, the population based Malmö Diet and Cancer Cardiovascular Cohort (MDC-CC) [14], which was designed to investigate the epidemiology of carotid artery disease. From a community-based prospective epidemiologic cohort of 28,449 persons enrolled between 1991 and 1996, 6,103 persons were randomly selected to participate in the MDC-CC. Whole blood samples for DNA extraction was obtained from 5445 subjects and genotypes for the main SNP rs10860812 were obtained from 5320 of these subjects. In the MDC-CC, BP was measured using a mercury-column sphygmomanometer after 10 minutes of rest in the supine position. Cardiovascular events (fatal and non-fatal stroke, fatal and non-fatal myocardial infarction and other cardiovascular deaths) were recorded during follow-up using national and local registers. Follow-up extended until December 31^st^ 2005. Intima-media thickness (IMT) of the carotid artery was measured using 2D B-mode ultrasound as described previously [15]. The mean IMT of a 10 mm section of the common carotid artery (IMT_mean_) and the maximum IMT of the carotid bulb (IMT_max_) was recorded.

Characteristics of the grade 2 hypertension patients (NORDIL) and the population based control sample (MDC-CC) are shown in Table 1.

**Table 1:** Characteristics of study subjects.

Characteristic	NORDIL	MDC-CC	MPP base	MPP followup
No. of individuals	5205	5320	16537	16537
Age (years±SD)	60.2±6.6	57.4±5.9	45.6±6.9	69.1±5.5
Female sex (%)	50.2	57.7	35.8	35.8
Antihypertensive treatment (%)	100*	16.7	4.5	38.4
Smoker (%)	20.6	27.7	36.9	17.5
Systolic blood pressure (mmHg±SD)	172.6±18.9	141.3±19.1	127.7±14.4	145.1±20.0
Diastolic blood pressure (mmHg±SD)	103.1±7.1	87.0±9.5	84.2±8.8	83.5±10.5
BMI (kg/m^2^±SD)	28.1±4.4	25.9±4	24.3±3.4	27.2±4.1
Glucose (mmol/l±SD)	5.32±1.56	5.19±1.42	4.90±0.75	5.85±1.4
Cholesterol (mmol/l±SD)	6.31±1.17	6.17±1.09	5.63±1.0	5.59±1.1

BMI, body mass index; SD, standard deviation. *all NORDIL patients had grade 2 hypertension and received treatment either prior to or during the trial.

### The Malmö Preventive Project

33,346 individuals from the city of Malmö (22,400 men and 10902 women) with a mean age of 49 years participated in a health study, the Malmö Preventive Project (MPP), during 1974–1992 (attendance rate 71%) [16]. The participants underwent a physical examination at the onset of the study with measurement of BP. Of those 33,346 participants, 5486 individuals were lost from follow-up (4931 died and 551 were lost from other reasons). 18,240 individuals participated in a re-screening visit during 2002–2006 (73% of the invited individuals) for renewed physical examination and BP determination. Of the 18,240 attending individuals, 1703 were excluded from the present investigation since clinical information or DNA was lacking. Thus, 16537 individuals were included for sequential replication. BP was monitored in different ways at the two occasions. At the onset of the study, a first BP reading was taken after 1 minute of rest in the supine position. A second BP reading was taken in the upright position after 1 minute of standing. The procedure was repeated after a 10 minute rest in the supine position. The average BP of subjects with at least three valid measurements was included in the present study. At the re-screening, BP was determined twice in the supine position and the average BP of individuals with two valid measurements was included in the present study.

Characteristics of the MPP study subjects are shown in Table 1.

### Genotyping

DNA was extracted from granulocyte or buffy coat suspensions, maintained at –80°C from the time of enrolment. Samples were thawed rapidly at 37°C and 200 µL aliquots were subjected to QiaAmp mini-preps in 96-well format (Qiagen) according to the manufacturer’s instructions. SNPs rs893881, rs10060812, rs2269772, rs4684243, rs4981504 and rs6891143 were genotyped using 2.5 ng of DNA on the 7900HT instrument using TaqMan SNP Genotyping Assays (Applied Biosystems) in a total reaction volume of 6 µL in 384-well microtiter plates, according to the manufacturer’s instructions.

### Animals

Adult C57Bl/6 mice were kept in groups at the Laboratory for Experimental Biomedicine at University of Gothenburg in a 12 h day/12 h night light cycle with food and water administered *ad libitum* in a temperature- and humidity-controlled room. All animal experiments were approved by the animal research ethics committee in Gothenburg, Sweden.

### Isolation of Microvascular Fragments

Microvascular fragments were isolated from adult C57/Bl6 mouse brain and kidney. Brains or kidneys were dissected out, minced into pieces and digested with 5 mg Collagenase A (Roche Diagnostics GmbH, Mannheim, Germany) dissolved in Hanks’ balanced salt solution (HBSS, Invitrogen AB, Lidingö, Sweden) including 1% BSA and 100U DNase at 37°C for 15 min with gentle agitation. The tissue was then gently pressed through a 100 µm cell strainer (Falcon, BD Biosciences, Stockholm, Sweden). Cells were washed out from the strainer in 2 ml of HBSS/1%BSA/100U DNase, pelleted at 200 g for 5 min, suspended in 1.5 ml HBSS/1% BSA/100U DNase, and again pelleted and resuspended. Rat anti-PECAM (BD PharMingen, San Diego, CA, USA) antibody (Ab) -coated magnetic beads (Dynabeads M-450, sheep anti-Rat IgG, Dynal A.S., Oslo, Norway) were added, and after incubation at 4°C for 30 min with gentle agitation, microvascular fragments were isolated with a magnetic particle concentrator (MPC, Dynal) and washed three times with HBSS/1% BSA.

### qPCR

mRNA expression levels were determined using SYBR Green quantitative qPCR (95°, 55°, 72°, 40 cycles) on a 7900HT instrument (Applied Biosystems) using the following primers: Adcy4, 5′-CTTTGGGTGGCTTCTCTCTG-3′ and 5′-ATGGCGTACACGGTGAAGAT-3′; Gpr116, 5′-AAGACATGAGATCGCCAAGG-3′ and 5′-TTGGCCTCAGTAGCTCTTCC-3′; Fgd5, 5′-GCTCAGGAGCTGCTGTCTTC-3′ and 5′-CCCTGGTGAAGGTCACAGATA-3′; Arap3, 5′-GACTGAGCCCAATCTTCTGG-3′ and 5′-TCGCCTGAGAACTATCTGGA-3′; Itga3, 5′-TTGAGGATATGTGGCTTGGA-3′ and 5′-ATGCCGGTCTGCAAGTAGTC-3′; Dram, 5′-GACACAGGAACAACTCCTCCA-3′ and 5′-AACGGGAGTGCTGAAGTAGC-3′; Nebl, 5′-ATGTTTCCACTGCGAGGTCT-3′ and 5′-TGCAGTTCACTTTGCTGCTT-3′. Expression of Gapdh and Tie2 were measured using TaqMan assays under standard cycling conditions (Mm99999915_g1 and Mm00443242_m1, Applied Biosystems). Relative expression levels were determined using the standard curve method [17].

### Bioinformatical Identification of Candidate Hypertension SNPs

Tab-delimited text files with hypertension association statistics for 469,557 SNPs were obtained from the WTCCC [2]. A list of 71 genes predicted to be specifically expressed in the microvascular endothelium were obtained from a recently published study [12]. Reciprocal human orthologs and genomic coordinates for these genes were determined using ENSEMBL and the UCSC browser [18], [19]. For each gene region, here defined as the start of the first exon until the end of the last exon, the SNP with the lowest trend P-value in the WTCCC study was identified (Table 2).

**Table 2:** SNPs in the WTCCC hypertension study mapped onto endothelial-specific genes.

Gene	Chr	Best SNP	*P*_add_	SNPs in gene	Gene	Chr	Best SNP	*P*_add_	SNPs in gene
ADCY4	14	*rs4981504*	4.10E-04	3	NP_061174	1	rs1175645	1.60E-01	18
GPR116	6	*rs2021916*	5.40E-04	43	C20orf160	20	rs6061095	1.60E-01	1
FGD5	3	*rs4684243*	8.10E-04	23	PSCD3	7	rs7777433	1.60E-01	7
ARAP3	5	*rs6891143*	1.00E-03	5	EPAS1	2	rs10191091	1.80E-01	23
ITGA3	17	*rs2269772*	1.50E-03	5	SMAD6	15	rs16950159	1.80E-01	12
DRAM	12	*rs10860812*	1.80E-03	7	TIE	1	rs1467809	2.30E-01	1
PTPRB	12	rs1867003	3.50E-03	42	ROBO4	11	rs12823	2.40E-01	3
SLCO2A1	3	rs10935089	7.70E-03	13	SLC43A3	11	rs3851116	2.40E-01	2
NOTCH4	6	rs415929	1.10E-02	8	NP_938019	4	rs17036363	2.40E-01	4
TCF21	6	rs3734281	1.30E-02	3	TENC1	12	rs2364153	2.50E-01	3
PECAM1	17	rs8074241	1.40E-02	14	PLTP	20	rs1736493	2.50E-01	2
CALCRL	2	rs840599	1.40E-02	22	KDR	4	rs17085310	2.80E-01	5
NP_631958	4	rs4365784	1.40E-02	4	SOX13	1	rs12092337	2.90E-01	3
NTN4	12	rs7961560	1.40E-02	19	ACVRL1	12	rs7956340	3.30E-01	3
ERG	21	rs2836431	1.70E-02	84	EPHB4	7	rs314313	3.30E-01	1
PTPRM	18	rs12457610	1.90E-02	171	LATS2	13	rs11841745	3.30E-01	19
RASIP1	19	rs2638283	2.20E-02	2	VEGF	6	rs3025047	3.50E-01	2
EHD4	15	rs1002774	2.50E-02	12	CCBP2	3	rs6763528	3.70E-01	4
Q8IW82	5	rs6882738	3.40E-02	14	ICAM2	17	rs4141180	4.10E-01	1
ITGA8	10	rs17137406	3.40E-02	65	NP_940873	19	rs1019757	4.60E-01	2
NRP1	10	rs870087	3.60E-02	45	CASKIN2	17	rs4789205	4.60E-01	3
COL4A3	2	rs11677877	3.90E-02	44	ESAM	11	rs7928640	6.40E-01	3
ELTD1	1	rs2035727	4.20E-02	15	STARD9	15	rs16957063	6.50E-01	4
NUMB	14	rs7148830	4.70E-02	18	HSPA12B	20	rs3088007	7.60E-01	1
NPR3	5	rs696833	5.10E-02	18	SDPR	2	rs4280394	8.00E-01	1
PALD	10	rs16927685	6.00E-02	32	ENG	9	rs11792480	8.10E-01	2
AGER	6	rs1035798	7.60E-02	1	ICAM1	19	n/a	n/a	0
MYO1B	2	rs897196	8.30E-02	35	CLDN5	22	n/a	n/a	0
CBFA2T3	16	rs519507	8.90E-02	6	Q8TAY7	1	n/a	n/a	0
DTR	5	rs4150212	9.30E-02	5	EGFL7	9	n/a	n/a	0
ENTPD1	10	rs10882664	9.50E-02	18	SLC9A3R2	16	n/a	n/a	0
MMRN2	10	rs12416136	1.20E-01	1	NP_078855	1	n/a	n/a	0
NP_079106	5	rs27059	1.40E-01	15	RAMP2	17	n/a	n/a	0
SOX7	8	rs7009920	1,50E-01	2	HIG2_HUMAN	7	n/a	n/a	0
CDH5	16	rs8051913	1.50E-01	6	NP_115724	1	n/a	n/a	0
LRRK1	15	rs7163635	1.60E-01	4					

SNPs selected for independent replication are indicated in italic. SNP counts refers to coverage on the Affymetrix 500K array.

### Co-localization of SNPs Associated with Hypertension or Blood Pressure in Meta-GWAS with EC-specific Genes

549 SNPs with P<10^−5^ based on data from 18 GWAS or meta-GWAS on hypertension or blood pressure were extracted from the PheGenI database (http://www.ncbi.nlm.nih.gov/gap/PheGenI) [2], [17]–[33]. 670 putative associated genes were within 100 kb these SNPs, and these were evaluated for overrepresentation of EC-specific genes (71 gene list) using Fisher’s exact test.

### Statistical Analysis

We assumed an additive model of inheritance and calculated allelic odds ratios (OR) and 95% confidence intervals (95% CI) for each SNP in relation to the main dependent variable (belonging to the grade 2 hypertension case group or belonging to the population controls) and to the secondary outcome variable (presence of cardiovascular events during follow-up in NORDIL or MDC-CC or no cardiovascular event during follow-up in NORDIL or MDC-CC) using crude and multivariate adjusted logistic regression. Continuous variables were related to rs10860812 using linear regression assuming an additive model of inheritance. Deviation from Hardy-Weinberg equilibrium was evaluated with a χ^2^ test using Levene’s method (1949).

## Results

### Putative Hypertension-associated SNPs in Endothelial-specific Genes

Since the endothelium has a functional role in BP regulation, we tested the hypothesis that genetic variation in genes expressed specifically in the endothelium may influence hypertension development. SNPs from the published WTCCC GWA analysis for human hypertension [2] were mapped onto a list of 71 endothelial genes obtained from a published study, where putative endothelial-specific genes were identified through analysis of a large mouse microarray compendium [12]. For each gene region, the SNP with the strongest association to hypertension was identified (additive model). In consistency with our hypothesis, the number of moderately significant SNPs (*P*<0.005) was significantly larger among the set of EC-specific genes compared to remaining genes (10% v.s. 4%, *P* = 0.03, Fisher’s exact test). Genes were ranked according to best *P*-value and SNPs in the top six candidates were selected for replication in an independent material. Association *P*-values in the WTCCC study for these SNPs were in the range of 1.8×10^−3^ to 4.1×10^−4^ (Table 2, italic). In addition, a SNP in the NEBL gene was included for validation due to its relatively strong association in the WTCCC (rs893881, *P*_add_ = 1.5×10^−5^). NEBL is selectively expressed in endothelial cells and was highly ranked in the original analysis of EC-specific gene expression [12], but did not qualify for the 71-gene list due to additional strong expression in the heart.

**Figure 2:**
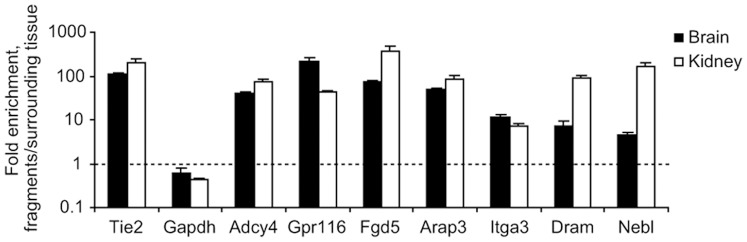
Differential mRNA expression in CD31+ microvascular fragments. Microvascular fragments were isolated from mouse tissues using anti-CD31 (PECAM)-coated magnetic beads. Reverse transcription was performed using equal amounts of RNA and expression levels were assessed using real-time quantitative PCR. The graph shows the ratio between expression in microvascular fragments and the remaining tissue fraction for all genes included in the study. Gapdh and an established endothelial marker, Tie2, were included for comparison. Error bars represent SEM (n = 4, *P*<0.005 in all cases using Student’s t-test).

To confirm expression of these genes in the vasculature, microvascular fragments were isolated from mouse brain and kidney using anti-CD31 (PECAM)-coated magnetic beads. Expression in vascular fragments vs. surrounding tissue was subsequently determined using real-time quantitative PCR. All genes were found to be significantly enriched in CD31+ fractions from both tissues (*P*<0.005) and all except one had >70-fold enrichment in at least one of the tissues (Figure 2). Gapdh was not differentially expressed, while Tie2, included as a positive control, was strongly enriched in CD31+ fractions from both tissues.

### The DRAM Locus and Hypertension

An independent evaluation of the above selected SNPs was performed in 5205 hypertensive patients and 5320 population based controls (NORDIL *vs.* MDC-CC). Genotyping was successful (average genotyping success rate 98%) for six of the seven SNPs selected for validation, while one SNP (rs2021916, GPR116 locus) did not pass quality control. For all tested SNPs, allele frequencies were fairly similar in the WTCCC study and the replication studies, and Hardy-Weinberg expectations were satisfied in the population control group (*P*>0.15). The power to show an association to hypertension in the case-control material (NORDIL+MCD-CC), assuming additive model odds ratios of 1.05, 1.10 and 1.15 was 21%, 60% and 90%, respectively.

One common variant (rs10860812) in the DRAM locus showed significant association with hypertension (Table 3), whereas none of the other SNPs were significantly associated. After applying a Bonferroni correction of 6 to account for all tested loci, this variant was still significant at the P<0.05 level. The minor allele (A) showed a protective effect (OR = 0.93; 95% CI 0.88–0.98 per A-allele; *P* = 0.008), the directionality of which is in concordance with the association that was observed in the WTCCC GWA study. The protective effect remained significant (OR = 0.93; 95% CI 0.87–0.99 per A-allele; *P* = 0.01) after adjustment for age, sex, body mass index, diabetes, smoking, plasma cholesterol, and previous cardiovascular disease. Exclusion of subjects on antihypertensive therapy in the control group (n = 888) had a small positive effect on the strength of the association (OR = 0.93, 95% CI 0.87–0.98 per A-allele; P = 0.007). We performed interaction tests between the DRAM rs10860812 and each of the 5 other SNPs with hypertension as the outcome. There was no significant interaction between the rs10860812 and any of the 5 other SNPs (P = 0.159–0.887).

**Table 3:** Association between hypertension and SNPs in the study in WTCCC and NORDIL/MDC-CC.

SNP					WTCCC hypertension study						
			Allele		Cases			Controls			
ID	Chr.	Locus	Major	Minor	AA	Aa	aa	AA	Aa	aa	*P*_add_
rs4981504	14q11	ADCY4	G	A	1092 (56.0)	734 (37.7)	123 (6.3)	1783 (60.7)	1014 (34.5)	141 (4.8)	4.1E-04
rs4684243	3p25	FGD5	A	T	988 (50.6)	787 (40.3)	177 (9.1)	1609 (54.8)	1122 (38.2)	206 (7.0)	8.1E-04
rs6891143	5q31	CENTD3	C	T	1368 (70.3)	531 (27.3	48 (2.5)	2186 (74.5)	695 (23.7)	55 (1.9)	1.0E-03
rs2269772	17q21	ITGA3	G	A	1682 (86.2)	261 (13.4)	8 (0.4)	2438 (83.0)	474 (16.1)	24 (0.8)	1.5E-03
rs10860812	12q23	DRAM	G	A	695 (35.7)	908 (46.6)	344 (17.7)	939 (32.1)	1387 (47.4)	602 (20.6)	1.8E-03
rs893881	10p12	NEBL	T	C	761 (39.6)	920 (47.8)	242 (12.6)	1000 (34.3)	1446 (49.7)	466 (16.0)	1.5E-05
SNP					NORDIL/MDC-CC replication study						
			Allele		Cases			Controls			
ID	Chr.	Locus	Major	Minor	AA	Aa	aa	AA	Aa	Aa	*P*_add_
rs4981504	14q11	ADCY4	G	A	3349 (64.3)	1649 (31.7)	211 (4.1)	3444 (64.9)	1659 (31.3)	204 (3.8)	4.6E-01
rs4684243	3p25	FGD5	A	T	2737 (52.7)	2041 (39.3)	418 (8.0)	2827 (53.3)	2113 (39.8)	367 (6.9)	1.6E-01
rs6891143	5q31	CENTD3	C	T	3464 (66.4)	1561 (29.9)	188 (3.6)	3593 (67.6)	1539 (28.9)	187 (3.5)	2.7E-01
rs2269772	17q21	ITGA3	G	A	4246 (81.6)	921 (17.7)	39 (0.7)	4375 (82.5)	880 (16.6)	46 (0.9)	2.9E-01
rs10860812	12q23	DRAM	G	A	1662 (31.9)	2521 (48.4)	1022 (19.6)	1553 (29.2)	2674 (50.3)	1093 (20.5)	7.8E-03
rs893881	10p12	NEBL	T	C	2055 (39.5)	2462 (47.3)	691 (13.3)	2118 (40.0)	2449 (46.2)	730 (13.8)	9.9E-01

*P*-values were calculated assuming an additive model. Figures within parentheses are percentages.

### Secondary Analyses of DRAM Locus in the MDC-CC Study

Secondary analyses of rs10860812 (additive models) in relation to the continuous BP variable in the MDC-CC population sample did not reach significance, although results pointed toward a protective effect for the A-allele (β-coefficient ± SEM) (−0.425±0.374 mmHg per A-allele, P = 0.26 for systolic BP and −0.200±0.187 mmHg per A-allele, P = 0.29 for diastolic BP). The risk of incident cardiovascular disease during follow-up (n = 894 cardiovascular events in MDC-CC+NORDIL) was reduced in carriers of the rs10860812 A-allele (OR = 0.90, 95% CI 0.82–1.0 per A-allele; P = 0.04). Finally, in patients from the MDC-CC study, where IMT was measured, the rs10860812 A-allele tended to be associated with lower (β-coefficient ± SEM) IMT_max_ (−0.028±0.015 mm per A-allele; P = 0.06) and IMT_mean_ (−0.006±0.003 mm per A-allele; P = 0.07).

### Secondary Analyses of DRAM Locus in the MPP Study

The rs10860812 variant was further evaluated (additive models) in relation to the continuous BP variable in the MPP study comprising 16537 individuals. There was no association between the rs10860812 variant and systolic or diastolic blood pressure neither at the MPP study baseline exam (β-coefficient ± SEM) (0.064±0.158 mmHg per A-allele, *P* = 0.688 for systolic BP and −0.068±0.097 mmHg per A-allele, *P* = 0.486 for diastolic BP) or at re-screening 15–25 years later (0.032±0.219 mmHg per A-allele, *P* = 0.883 for systolic BP and −0.121±0.116 mmHg per A-allele, *P* = 0.297 for diastolic BP). One possible confounding factor is that pharmacological treatment of hypertensive subjects in the study lowers the observed BP thus reducing the statistical power [20]. However, similar results were obtained when diastolic (+10 mmHg) and systolic BP (+15 mmHg) were corrected in subjects on antihypertensive treatment (data not shown), as described in [6]. Moreover, the risk of incident cardiovascular disease during follow-up was not different in carriers of the rs10860812 A-allele, compared to the control population (data not shown).

### Co-localization of EC-specific Genes and SNPs from Meta-GWAS

The lack of significant associations in our study suggests that variants in EC-specific genes may not be associated with hypertension or blood pressure more often than random genes. However, the publication of large meta-GWAS has expanded the repertoire of SNPs associated with those phenotypes, raising the possibility that newly discovered SNPs co-localize with EC-specific genes. To assess this possibility, we tested the 71 EC-specific genes against a recent catalogue of SNPs associated with hypertension or blood pressure [21], comprising a total of 18 GWAS or meta-GWAS [1], [3], [5], [6], [22]–[35]. A set of 529 SNPs with association *P*<10^−5^ were downloaded from the genotype phenotype integrator (PheGenI) at NCBI (http://www.ncbi.nlm.nih.gov/gap/PheGenI), and mapped against the genomic locations of the 71 EC-specific genes. EC-specific genes were not significantly over-represented among 670 genes located within 100 kb of those SNPs (5 EC-specific genes located within 100 kb of associated SNPs, *P = *0.10, Fisher’s exact test).

## Discussion

Rare high-penetrance or common weakly penetrant genetic variants are not readily detected in GWA studies, opening up for alternative approaches that maintain the genome-wide perspective but reduce multiple testing. Here, we assess if genetic variants in endothelial-specific genes are associated with hypertension. The SNPs were selected for validation based on 1) endothelium-specific expression of the gene locus and 2) an association to hypertension that was observed in the WTCCC GWA study, although at a *P*-value below genome-wide significance. The selection process can thus be described as an intermediate between a candidate gene approach and a genome-wide approach.

Our case control study was substantially larger and our cases had a strictly defined and more severe clinical hypertension (grade 2) compared to the WTCCC, suggesting a greater power as the genetic contribution is likely to be larger in this case. Similar to the WTCCC, our control group was population based and thus included patients with hypertension. Our estimate of the genetic effect on risk therefore has relevance at the population level, but this may also have reduced the power in our study. To further validate effects on BP, SNPs were re-evaluated in relation to the continuous BP variable in the population based MPP study.

Our first replication suggested an association between a SNP (rs10860812) in the DRAM locus on chromosome 12q23 and hypertension. DRAM is a regulator of autophagy that plays a critical role in apoptosis [36]. Although open to question, the involvement of apoptosis in hypertension-related vascular remodeling has been suggested [37]. The minor allele (A) of this SNP was concluded to have a protective effect and this is consistent with results from the WTCCC study. Secondary analyses revealed an association with incident cardiovascular events and borderline significant associations with IMT_max_ and IMT_mean_. However, evaluation of rs10860812 in relation to the continuous blood pressure variable in the MDC-CC population study and sequentially in the larger MPP study did not show association with BP. Moreover, there was no association of rs10860812 with cardiovascular disease in the MPP study.

Recent meta-analyses have to some part overcome the problems of the early hypertension GWAS, and 29 loci are now robustly associated with blood pressure and hypertension [3]. The EC-specific gene NRP3 is located near one of the 29 validated loci, rs1173771, and encodes a receptor for natriuretic peptides that are implicated in the maintenance of blood pressure [38], supporting a causal role for the endothelium. A large proportion of blood pressure heritability remains unknown [3], and alternative methods such as the one we describe could help identify additional loci. However, our replication studies of moderately significant SNPs from the WTCCC study did not support a connection between genetic variation in the endothelium and hypertension. Similarly, the expanded investigation of 529 potential SNPs from the PheGenI catalogue failed to show significant overlap with EC-specific genes.

In conclusion, our study does not support that genetic variation in genes expressed in endothelium plays a major role in the development of hypertension. The result further underscores the importance of rigorous validation of genetic associations in large and independent populations.
